# Nanonutraceuticals Delivery

**DOI:** 10.3390/nano11082031

**Published:** 2021-08-10

**Authors:** Luciana Dini, Cristian Vergallo

**Affiliations:** 1 Department of Biology and Biotechnology C. Darwin, Sapienza University of Rome, Piazzale A. Moro 5, 00185 Rome, Italy; 2 Interdepartmental Research Center on Nanotechnology Applied to Engineering of Sapienza (CNIS), University of Rome, Piazzale A. Moro 5, 00185 Rome, Italy; 3 CNR Nanotec, 73100 Lecce, Italy; 4 Institute of Higher Secondary Education “Presta-Columella”, Technical and Technological Sector “Agriculture, Agri-Food and Agro-Industry”, Via S. Pietro in Lama snc, 73100 Lecce, Italy; cristian.vergallo@istitutocolumella.it

Technological innovation, environmental sustainability, health, and wellness are the trajectories explored by current research to identify new strategies for a general improvement of human quality of life. Nanotechnology is a mandatory crossing point since different nanomaterials represent a bridge between the growing and unstoppable demand for high safety for human health and respect and protection for the environment [1]. Among the many nanotechnology applications, there is an effort in the production of nanoscale materials for the food industry by the characterization, fabrication, and manipulation of structures, devices, or materials having at least one dimension not exceeding 100 nm [2]. The small size of nanomaterials makes them ideal to improve food quality, shelf life, safety, cost, and nutritional benefits [3]. In addition to research on food packaging, which is still occupying most of the research in the food field, a growing interest is now focusing on nanosized food ingredients, also known as nanonutraceuticals [4,5,6]. These products increase the functionality or bioavailability of nutrients, showing beneficial effects on human health by preventing the onset of several pathologies, including cancer and several cardiovascular and neurodegenerative disorders. However, 70% of compounds with possible usage as nutraceuticals have poor aqueous solubility leading to difficulty crossing cell membranes [7]. This problem is addressed by using different formulation approaches such as particle size reduction (nano-sizing) and the use of the amorphous form and lipid-based drug delivery systems, albeit with specific drawbacks that must always be taken into consideration. The most recent investigations aimed to exploit nanotechnology for overcoming the main limitation as for example poor aqueous solubility of most of the nutraceuticals, is discussed here. The present Special Issue is devoted to compiling several papers, and it is composed of 3 research articles and 6 reviews. This new nanotechnology aims to provide further knowledge in the field of application of nanomaterial-based carriers involved in nutraceutical delivery through original articles focused on the development of innovative synthesis processes, as well as in vitro and in vivo studies assessing the biological effects and/or physical and chemical properties of them.

The common theme running through the various papers is the use of nano-bio-based materials as natural drug compounds or as drug delivery system of nutraceuticals for the vital biomedical applications, such as for tumours, cardiovascular, and neurological disorders, etc. Original research articles and review papers highlight theoretical and practical concepts and protocols for nanomaterials applications.

The papers that make up this special issue discuss a broad spectrum of different nanomaterials, such as nanochitosan [8] vegetable oil [9], oil in water, and nanoemulsion/nanoemulgel [10,11], ufasomes [12], and vitamin drug delivery [11,13].

The work of Paolino et al. [14] points out the potential efficacy of nutraceuticals and, due to their limiting features, how nanotechnology is a revolutionary empowerment of the beneficial properties on human health. Special addressing is dedicated to the new frontier of nanotechnology as an innovative tool in supplementary food. The authors discuss the most recent approaches of nanotechnology to overcome the poor bioavailability of nutraceuticals, highlighting the pros and cons of several nanodelivery systems. Nutraceuticals include dietary supplements and functional foods [4] that give benefit to human health by positively affecting the immune system and strengthening it in these turbulent days of the COVID-19 pandemic [7]. The body’s immune responses to nanonutraceuticals are discussed in relation to vitamins (C, D, E, B12, folic acid), minerals (Zn, Fe, Se), antioxidants (carotenoids, coenzyme Q10, polyphenols, curcumin, omega-3 fatty acids), and probiotics [15]. In particular, Panzarini et al. [7] discuss the potential effect of curcumin on glia cells and its therapeutic and protective role in central-nervous-system-related disorders. The curcumin nanoconstruct can increase curcumin stability and solubility, in vivo uptake and allows it to overcome the blood-brain barrier (BBB) obstacle, which is particularly important in the presence of brain pathologies. Of great interest, since it has functional features that could be applied for crossing the BBB, is the use of an exosome-based delivery system, whose therapeutic potential seems to be remarkable also in favouring the passage of curcumin.

A decidedly new experimental approach is the administration of vitamin B12 (vit. B12) using nanomaterials as reviewed by Fidaleo et al. [13]. Vit. B12 is an essential micronutrient, and it is crucial for life. Several papers report the role of vit. B12 deficiency in many metabolic dysfunctions and the need to supply it for survival in inborn errors of vit. B12 metabolism. In the review is discussed how nanocarriers can improve the therapy and supplementation of the vitamin and reduce possible side effects and limits.

The current fashionable term of “nutraceutical” has been coined some time ago combining the words “nutrition” and “pharmaceutical” for indicating a natural product eliciting a medical or health benefit [16] and thus the coexistence of two different approaches. In this context, Cristiano et al. [12] introduce the term “the super functional food” referring to the natural product oleuropein. This new term is linked to the concepts of environmental impact and circular economy; oleuropein is extracted from the leaves of the olive tree thus making them a source of nutraceutical product instead of waste material. A similar careful look at both environmental impact and circular economy is present in the work of Sivanesan et al. [8] in which nanochitosan is converted from exoskeletal waste to a versatile nutraceutical, being the second, after cellulose, most abundant organic polymer. In its deacetylated form, chitosan is nowadays interesting material for medical use. The progress of nanochitosan from benchtop to bedside is extensively evaluated in the paper, suggesting the inclusion of nanotechnology in the chitosan research for breaking down any possible limitations in production and applications. Unbeknownst to us, nutraceuticals are typically consumed as part of a normal human diet and are usually found in foods, including vegetable oils, albeit at low levels and varying compositions. Therefore, it is difficult to control the type, quantity, and frequency of their ingestion by individuals. Nanoformulations about vegetable oil-based bioactive compounds with nutraceutical properties are useful for overcoming these issues while improving the uptake, absorption, and bioavailability in the body [9]. For this Special Issue, Vergallo [9] reviews papers on such nanoformulations, especially those relevant for health benefits, diseases prevention and management, as well as molecules extracted from vegetable oils that enhance the efficacy of the drug, recovered through bibliographic databases setting a time frame from January 2000 to April 2020 (about 1758 records).

This special issue is completed with two research articles. In the first paper [10] the in vitro activity on cancer cells of resveratrol-loaded nanoemulsion is investigated and in the second [11] the formulation design and stability of topical delivery of retinyl palmitate are evaluated. Rinaldi et al. [10] present their data showing oil-in-water nanoemulsions ideal candidates for resveratrol encapsulation, due to the significant decrease in cell viability of bladder T24 cancer cells. In their article, Algahtani et al. [11] report a novel process to obtain the retinyl palmitate loaded nanoemulgel, that exhibited significant improvement in skin permeability after topical application.

As Editors of this Special Issue entitled “Nanonutraceuticals Delivery”, we are confident that it will contribute to the research interest in this broad field, that includes nanonutraceuticals production and delivery, dietary nanosupplements, food nanoadditives, and nanomaterials, functional nanofoods, nanoencapsulation of nutraceuticals and nutrients, bio-based nanoformulations of nutraceuticals, pharmaceutical-grade, and standardized nanonutrients, etc., stimulating researchers to explore new strategies for the improvement of large-scale synthesis and economic viability of the proposed nanomaterials with an increased positive impact on human health.

We are well aware that the rapid development in this multidisciplinary research field cannot be fully reflected in this special issue, but we are confident that it can encourage multidisciplinary research on bio-based nanomaterials produced by green processes.
